# Exploring the legacy of Central European historical winter wheat landraces

**DOI:** 10.1038/s41598-021-03261-4

**Published:** 2021-12-13

**Authors:** András Cseh, Péter Poczai, Tibor Kiss, Krisztina Balla, Zita Berki, Ádám Horváth, Csaba Kuti, Ildikó Karsai

**Affiliations:** 1 Agricultural Institute, Centre for Agricultural Research, ELKH, Martonvásár, 2462 Hungary; 2 Finnish Museum of Natural History, University of Helsinki, 00014 Helsinki, Finland; 3 Faculty of Biological and Environmental Sciences, University of Helsinki, 00014 Helsinki, Finland; 4 Centre for Research and Development, Food and Wine Center of Excellence, Eszterházy Károly Catholic University, Eger, Hungary

**Keywords:** Plant breeding, Plant domestication, Agricultural genetics

## Abstract

Historical wheat landraces are rich sources of genetic diversity offering untapped reservoirs for broadening the genetic base of modern varieties. Using a 20K SNP array, we investigated the accessible genetic diversity in a Central European bread wheat landrace collection with great drought, heat stress tolerance and higher tillering capacity. We discovered distinct differences in the number of average polymorphisms between landraces and modern wheat cultivars, and identified a set of novel rare alleles present at low frequencies in the landrace collection. The detected polymorphisms were unevenly distributed along the wheat genome, and polymorphic markers co-localized with genes of great agronomic importance. The geographical distribution of the inferred Bayesian clustering revealed six genetically homogenous ancestral groups among the collection, where the Central European core bared an admixed background originating from four ancestral groups. We evaluated the effective population sizes (*N*e) of the Central European collection and assessed changes in diversity over time, which revealed a dramatic ~ 97% genetic erosion between 1955 and 2015.

## Introduction

Wheat (*Triticum aestivum* L., AABBDD, 2n = 6x = 42) is traditionally one of the main food sources of humankind and modern cultivars provide 15% of calories consumed every day^1^. Despite its global impact on food security, domesticated wheat faces critical challenges generated by the changing climate. Climate change manifested in increased temperatures, drought or alteration in rainfall frequency and intensity is already affecting agriculture, posing a further barrier to efficient wheat production^2^.

Wheat landraces as traditional wheat varieties, may preserve a specific capacity to tolerate biotic and abiotic stresses, resulting in yield stability and an intermediate yield level under a low input agricultural system^3,4^. Valuable agricultural characteristics, e.g., stress tolerance and quality traits, can be readily introduced from landraces into new high-yielding wheat varieties in order to ensure food security for the rapidly growing population of the world.

Genetic variation provides the basis for crop adaptation in diverse environments. During the ‘green revolution’, wheat breeding focused on the development of high-yielding, disease-resistant wheat varieties with dwarfing genes that reduced the genetic diversity of modern elite varieties leaving a legacy of great variation behind in landraces. The wider genetic diversity within wheat landrace populations including agronomically advantageous morpho-physiological traits (e.g. traits regarding the root system) are responsible for the better adaptation to changing climatic conditions. Population genetic studies provided extensive evidence for the greater genetic variation in bread wheat landraces and highlighted them as excellent sources of unaccustomed alleles potentially useful for modern breeding^5–9^. Wheat improvement programs can greatly benefit from the diverse genetic background preserved in these populations to ensure food security and sustainable, climate-smart agriculture in the future^2,10^.

There are clear examples of how exploiting landraces to introduce novel yield or drought characteristics into modern wheat, have resulted in global economic impacts. For instance, the introduction of dwarfing genes (*Rht-B1b, Rht-D1b*) from the Japanese cultivar ‘Norin 10’ during the ‘green revolution’ led to spectacular increases in yield^11^. ‘Norin 10’ inherited these genes from the landrace ‘Shiro Daruma’. Another example is the old Hungarian wheat cultivar ‘Bánkúti 1201’ developed in the first half of the 20th century, which contributed to the significant diversity and several unique alleles of modern Hungarian wheat cultivars, known for their improved bread making quality parameters^12–14^.

The Central European wheat landrace collection including 199 accessions originated from six countries were collected during 1950–1960 and conserved in the Gene Bank collection of the Centre for Plant Diversity (NÖDIK) Tápiószele, Hungary. This important legacy of Hungarian wheat breeding represents a vast and largely untapped source of genetic diversity. These wheat landraces are generally tall, prone to lodging and collectively considered to be highly adaptable to the agro-ecological conditions of Central Europe. They have excellent drought and heat stress tolerance and stronger tillering ability under low nutrition input farming conditions^15,16^. As a potential source of useful loci to improve wheat stress tolerance and grain quality, it is essential to characterize the phenotypic and genotypic diversity present among the Central European landrace collection.

Modern marker-assisted selection (MAS) programs are based on genetic markers, but their use in wheat breeding was encumbered for a long time by the large genome size and the presence of three homoeologous genomes (ABD). The level of genetic diversity within a population can, however, be measured by single nucleotide polymorphism (SNP) genotyping arrays that offer the anticipated impetus to accelerate wheat breeding^17,18^. By using SNP genotyping, many lines can be cost-efficiently screened at an early stage making it possible to design more effective breeding programs. Such arrays and genotype calling algorithms can successfully identify SNPs across a broad range of wheat as it was demonstrated in populations with hexaploid- or tetraploid backgrounds, from landraces to modern varieties^19,20^. When SNPs are chosen from a limited panel, the ascertainment bias for chip data is introduced^21^. While SNP arrays are susceptible to such bias due to preselection of SNPs in small populations^21^, their low computing needs for downstream data processing, high call frequency, low error rate, and simplicity of use make them a desirable genotyping instrument. To prevent SNP ascertainment bias, whole genome sequencing should be utilized ideally. However, since small sample sizes affect allele frequency distributions, one may never be entirely free of SNP ascertainment bias^22^. The study by Chu et al.^23^ examined the influence of ascertainment bias on array-based SNP markers, which resulted in an underestimate of molecular diversity among wheat populations. Their study revealed that the marker system had a minor effect on the overall image of population structure and the accuracy of genome-wide predictions. They also noted that uncommon markers significantly improved prediction performance; this, along with the assumption that new diversity would most likely be uncommon, implies that minor allele frequencies should be carefully considered when designing a pre-breeding program. Other studies implied that ascertainment bias can also be corrected by incorporating maximum likelihood methods^24^, Bayesian estimations^25^ or haplotype statistics^1^.

High-power Genome-Wide Association Studies (GWAS) are also based on information gained from high-throughput SNP genotyping. GWAS enables the identification of markers linked to agronomically important genes and their transfer via MAS from landraces into modern cultivars. For GWAS analysis the population structure needs to be investigated to avoid false positive associations between phenotypes and markers^26^.

Here we explored the genetic structure, effective population size and the available genetic diversity in the Central and Eastern European bread wheat landraces by using a 20K SNP array. We then compared these landraces to modern wheat cultivars and to gain insight to changes in diversity over time.

## Results

### Novel polymorphisms among Central European landraces

To compare the variability of modern elite varieties and landraces, we genotyped the two collections using a 20K array containing 17,267 SNPs to identify polymorphisms across the 21 chromosomes of hexaploid wheat. Genotyping resulted in 15,808 high quality SNPs (Supplementary Table S1), while 1459 (8.44%) SNPs were trimmed from the final analysis during quality assessment. The selected 15,808 markers showed an average heterozygosity index (*H*) of 0.78, polymorphic information content (*PIC*) of 0.70, and had a discriminatory power (*D*) of 0.31 among all investigated accessions. Due to the possibility of ascertainment bias caused by the marker type and selection, we inferred haplotype blocks and matching alleles throughout the genome using chip-based markers^27^. This was accomplished by partitioning the bread wheat genome into 1984 regions with limited evidence of recombination and just a few common haplotypes. The average number of haplotype blocks per chromosome was 94.48, ranging from 5 to 178 and it was correlated with chromosome size (Supplementary Table S2). The average haplotype diversity (*H*_d_)^28^ was 0.46, similarly to the findings of Balfourier et al.^1^ using 4,506 accessions and 280,226 genic and intergenic SNPs. The reduced diversity reported when haplotypes are used may be due to the increased number of alleles, especially rare ones, as Balfourier et al.^1^ also noted. From the selected markers, 15,565 (98.46%) were polymorphic across the two collections considered together, while there were 15,121 (95.65%) and 15,357 (97.14%) polymorphic markers in landraces and modern varieties, respectively. Most of the detected markers (14,913; 94.33%) were polymorphic in both collections. Nucleotide diversity (*π*), defined as the number of nucleotide differences per site between two randomly chosen sequences, was estimated to be 2.6 × 10^−3^ in the entire collection. Landraces showed a greater value (2.5 × 10^-3^) compared to modern varieties (1.03 × 10^−3^) in line with previous estimates^29,30^.

**Figure 1:**
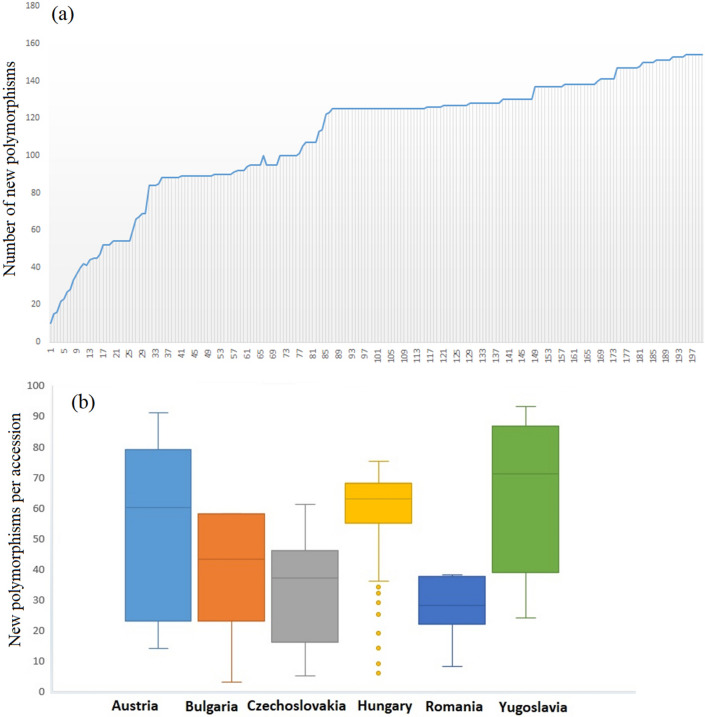
(**a**) Plot depicting the gradual incorporation of novel polymorphisms when more Central European accessions are added to the scores of modern lines. (**b**) Box and whisker plots depicting the amount of extra polymorphic markers introduced as each accession from each country was added to the modern varieties one by one. Outliers among Hungarian accessions are marked with yellow dots.

**Figure 2:**
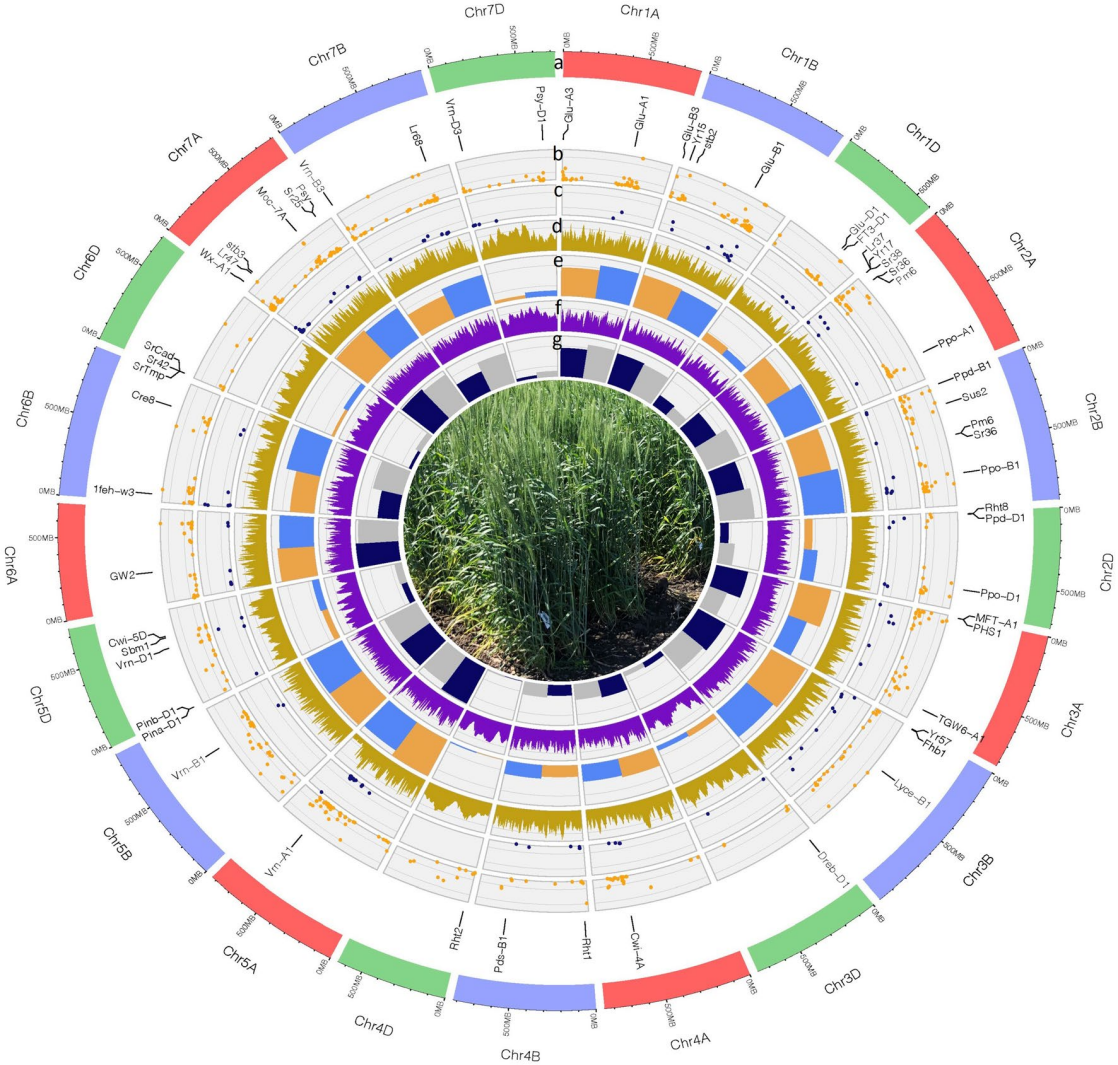
Circos diagram showing the physical map of twenty-one wheat chromosomes. (**a**) The physical scale (Mbp) of the A (*red*), B (*blue*) and D (*green*) genome chromosomes of wheat. Chromosomes are displayed by size proportioned bars in the outer circle. The position of genes encoding important agronomic traits are marked under the bars (*see text for further details*). (**b**) The chromosome position and frequency of polymorphic SNPs (*yellow dots*) found in Central European landraces compared to modern Western varieties. (**c**) The chromosome position and frequency of polymorphic SNPs (*dark blue dots*) found in landraces compared to modern varieties. (**d**) Nucleotide diversity (*π*) of Central European landraces compared to modern varieties in 3 Mb window intervals (*light brown*). (**e**) Bar chart showing the total number of SNPs compared to the ‘MV Ménrót’ reference in modern varieties (*orange*) and landraces (*light blue*). (**f**) Nucleotide diversity (*π*) of Central European landraces in 3 Mb window intervals (*purple*). (**g**) Marker density of polymorphic SNPs based on the ‘MV Ménrót’ reference in modern varieties (*grey*) and landraces (*dark blue*). Tracks are marked alphabetically (a-g) from top to bottom.

After determining that landraces contain a considerable number of polymorphisms, we were interested in investigating how many of these polymorphisms are unique to the Central European collection. We also assessed whether a geographical bias exists in this regard, resulting in some accessions being more polymorphic than others on a regional basis. Thus, the genotypic scores of Central European landraces were sequentially added to the scores of the accessions consisting of all modern varieties in order to determine the number of novel polymorphisms following Winfield et al.^20^ This cumulative addition revealed that the average number of novel polymorphisms is 82 (range 10–154) (Fig. 1a). The list of novel polymorphisms is available as Supplementary Table S3. The smallest number of novel polymorphisms (10) was present in ‘CLUJ50-650’ from Romania, while the addition of ‘Banja-Luka-6’ (former Yugoslavia), ‘Garljana’ (Bulgaria) and ‘Martonvásári-K118’ (Hungary) contributed 154 new polymorphisms to the modern varieties. The Central European wheat landraces harbored a collection of rare alleles present at low frequencies (< 0.05, 78%; Supplementary Table S3 and Fig. 2b). It is possible that these alleles might have had some selective advantage during breeding and improvement. A similar scenario was also reported in other landrace collections^31,32^. Only a few alleles (11%) were found at higher frequencies (> 0.15) among the novel polymorphisms. These alleles are likely responsible for the wide adaptation of the landraces, as shown by previous studies in wheat^6,33^, maize^34^ and rice^35^.

Overall, landraces originating from the Balkan area had the largest number of novel polymorphisms per accession (Fig. 1b). There was a distinct difference in the number of average novel polymorphisms on the basis of an East-West geographical gradient. When we added Central European landraces to the genotypic scores of modern elite cultivars, on average 449 (range 10–888) new polymorphisms were detected. This suggests that these landraces possessed many novel polymorphisms compared to modern varieties. Thus, the Central European collection could be a good source to improve the genetic diversity of the elite varieties.

### Polymorphic markers co-localize with agronomically important genes

The 20 K array was constructed from SNPs that were previously mapped in the wheat genome, enabling us to assess their distribution and allele frequencies among the 21 wheat chromosomes and seven homoeologous groups^19^ (Fig. 2a–c). The detected polymorphisms were unevenly distributed along the wheat genome, considering the size of the chromosomes using the estimates of Šafář et al.^36^. The number of polymorphisms was highest on the A-genome and lowest on the D-genome compared to the ‘Mv Ménrót’ (Martonvásár, Hungary) reference (Fig. 2e and Supplementary Table S4). In general, the A and B genomes were more diverse and showed more uniform distributions of polymorphisms across the genome than the D genome, in agreement with Akhunov et al.^30^ The average marker density was also the lowest in the D-genome compared to the others (Fig. 2f and Supplementary Table S4), in line with previous studies^37^. Chromosome 5A had the highest number of polymorphic SNPs, while 4D had the least. In general, a relatively high positive correlation was observed between the number of polymorphic SNPs and chromosome size. This has similarly been reported by Alipour et al.^32^.

A higher number of polymorphisms was concentrated on the homoeologous group 2 of landraces when the genetic origin of the accession was considered (Supplementary Table S4). In the case of group 4, 6 and 7, only chromosomes of the B and D genomes were more diverse in landraces compared to the modern elite varieties. On the contrary, the A genome from all homoeologous groups except 1 and 2 showed more polymorphisms in modern varieties. Mapping the positions of polymorphic markers along several important genes of great agronomic importance indicated that novel polymorphisms co-localize with these genes (Fig. 2 and Table 1). These included reduced height genes (*Rht1*, *Rht2*), photoperiod response genes (*Ppd-B1*, *Ppd-D1*), as well as several genes associated with disease resistance (*Lr*, *Sr*, *Yr*, *Pm*), suggesting that they might have provided the basis of selection during the breeding programs.

**Table 1:** List of agronomically important genes from Circos diagram.

Gene	Function	Chr
*Glu-A1*	Low-molecular-weight (LMW) glutenin	1A
*Glu-A3*	Low-molecular-weight (LMW) glutenin	1A
*Glu-B1*	Low-molecular-weight (LMW) glutenin	1B
*Glu-B3*	Low-molecular-weight (LMW) glutenin	1B
*stb2*	Septoria tritici blotch (STB) disiease resistance	1B
*Yr15*	Yellow rust broad disease resistance	1B
*Glu-D1*	Low-molecular-weight (LMW) glutenin	1D
*FT3-D1*	Flowering locus, T-Like Poaceae gene family, yield traits	1D
*Sr36/Pm6*	Stem rust resistance gene	2A
*Lr37/Yr17/Sr38*	Yellow rust broad disease resistance; multi disease resistance	2A
*Ppo-A1*	Polyphenol oxidase gene	2A
*Sus2*	Sucrose synthase gene	2B
*Sr36/Pm6*	Stem rust resistance gene	2B
*Ppo-D1*	Polyphenol oxidase gene	2D
*Ppd-D1*	Polyphenol oxidase gene	2D
*Rht8*	Reduced plant height	2D
*MFT-A1*	Flowering locus, T-Like Poaceae gene family, yield traits	3A
*PHS1*	Flowering locus, T-Like Poaceae gene family, yield traits	3A
*TGW6-A1*	Flowering locus, T-Like Poaceae gene family, yield traits	3A
*Fhb1*	Fusarium head blight resistance	3B
*Lyce-B1*	Lycopene gene	3B
*Yr57*	Yellow rust broad disease resistance	3B
*Dreb-D1*	Dehydration-responsive element binding (DREB) protein, drought tolerance	3D
*Rht-B1*	Reduced plant height	4B
*TaPds-B1*	Flowering locus, T-Like Poaceae gene family, yield traits	4B
*Rht1*	Reduced plant height	4B
*Rht2*	Reduced plant height	4D
*Vrn-A1*	Vernalization gene	5A
*Pinb-D1*	Grain hardness	5D
*Pina-D1*	Grain hardness	5D
*Sbm1*	Soil-borne wheat mosaic virus (SBCMV) resistance	5D
*Cwi-5D*	Flowering locus, T-Like Poaceae gene family, yield traits	5D
*Vrn-D1*	Vernalization gene	5D
*GW2*	Flowering locus, T-Like Poaceae gene family, yield traits	6A
*1feh-w3*	fructan exohydrolase, drought tolerance	6B
*Cre8*	Resistance loci against the cereal cyst nematode (CCN) Heterodera avenae	6B
*SrCad, Sr42, SrTmp*	Stem rust resistance gene	6D
*Psy/Sr25*	Carotenoid biosynthesis genes	7A
*Lr47*	Leaf rust resistance gene	7A
*stb3*	Septoria tritici blotch (STB) disiease resistance	7A
*Moc-7A*	Flowering locus, T-Like Poaceae gene family	7A
*Wx-A1*	Granule-bound starch synthase or waxy, Wx loci	7A
*Lr68*	Leaf rust resistance gene	7B
*Vrn-B3*	Vernalization gene	7B
*Psy-B1*	Carotenoid biosynthesis genes	7D
*Vrn-D3*	Vernalization gene	7D

The name of several genes of great agronomic importance^60,61^.

### Genetic and geographical structuring of wheat accessions

**Figure 3:**
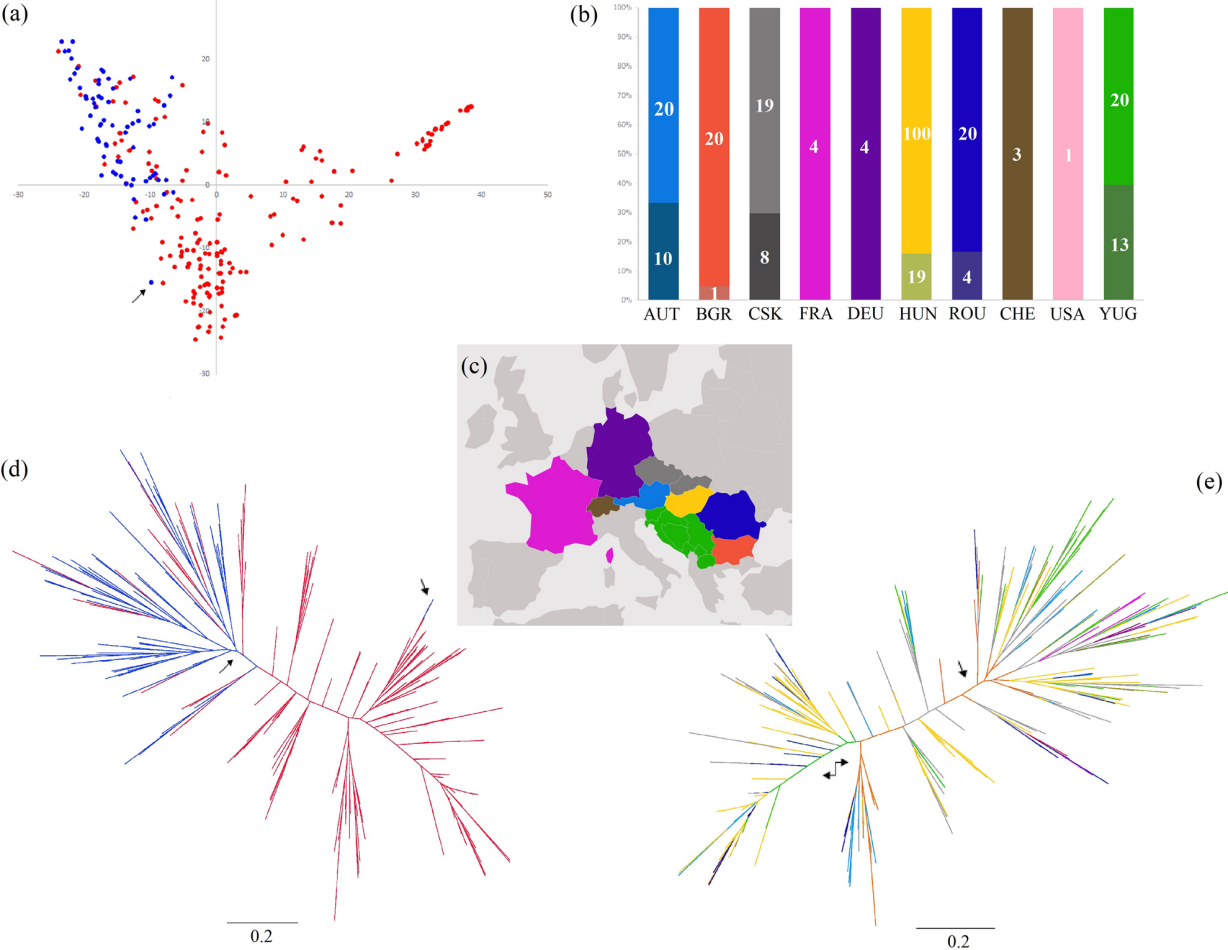
Genetic and geographical structuring of wheat accessions. (**a**) PCA plot showing the relationship between the accessions belonging to the landraces (*red dots*) and modern varieties (*blue dots*). The modern elite variety ‘Divana’ from Croatia closely grouped with the landraces (*black arrow*). (**b**) Using representative coloring the number of accessions is shown as bars for each region. Darker shades and inserted numbers indicate modern varieties. A total of 266 accessions were included in our study (see Supplementary Table S5). (**c**) The ten regions are marked on the map from which the wheat accessions were collected using the same coloring. (**d**) Unrooted maximum likelihood (ML) tree generated with IQ-Tree, with overlayed genetic origin of the accessions; branches representing modern varieties (*blue*) and landraces (*red*). The well-supported (bootstrap > 90%) division (*blue split*) of modern varieties is marked with a *black arrow*. The position of ‘Divana’ is indicated with the second *black arrow* pointing to the *blue* branch nested within the *red* group of landraces confirming its close affinity. (**e**) Unrooted ML tree with overlayed representative coloring (see **b** and **c**) corresponding to the country of origin of the accessions. Arrows indicate well-supported groupings (bootstrap > 90%) in both ML trees (**d** and **e**) while the rest of the nodes received weak signal (< 35%). A general time reversible nucleotide evolutionary model with direct base frequency counts was used to infer topologies. The trees are draw to scale with branch lengths measured in the number of substitutions per site. The scale bar represents 0.2 substitution per site.

The collection of winter wheat accessions were distinguished based on their chronological origin (landraces vs modern elite varieties) in order to allow further comparison between geographical and genotypic data (Fig. 3b,c and Supplementary Table S4). Principal component analysis (PCA), Bayesian cluster (STRUCTURE) and maximum likelihood (ML) tree analyses were conducted to determine the population structure of the wheat collection, using the set of 15,808 markers and after removing 285 SNPs with minor allele frequencies (< 0.01). We focused on the overall population structure, which was shown to remain less affected by ascertainment bias^23^. Resulting patterns (Fig. 3e) depicted close relationships and admixture within the accessions. This was also suggested by the low bootstrap values (< 35%) obtained for most of the resulting groups in the ML analysis, indicating a lack of consistent signal to cluster these accessions to fine-scale inner groups. This could be attributed to recombination or high divergence within a short timescale, resulting in relatively high homoplasy compared to the number of informative sites, ultimately making the signals too weak. These patterns could be expected with our wheat collection; however, we retained two well supported groups (> 90%) in the tree (Fig. 3e, indicated with arrows). Similar patterns were observed in the PCA analyses where both the first (PC1, 19.08%) and second component axis (PC2, 9.2%) explained very little at the regional level (Supplementary Fig. S1), but were informative when chronological origin of the collection was considered (Fig. 3a).

Overlaying the chronological origin of the accessions on the ML tree (Fig. 3d) and the PCA showed clear differentiation between the modern varieties and landraces. The PCA summarizes the dominant components of variation in the genomic data, showing the difference between sampled regions but also including the variation within groups of accessions, thus limiting the amount of between-population variation explained by the two principal component axes^38^. In this respect, the first component axis explained the genomic variation found in the modern varieties, while the second component axis explained the variation mostly found in the landraces, in accordance with the ML tree. Interestingly, the Austrian landraces grouped together with the modern varieties in the upper right corner of the PCA plot (Supplementary Fig. S1). Tightly grouped modern elite varieties were principally located at the upper right corner of the plot (Fig. 3a; marked with blue), while the landraces were evenly distributed among the two axes (marked with red). This division among the accessions was highly supported by high ML bootstrap values (>90%). The modern elite variety ‘Divana’ from the Adriatic region (Croatia) closely grouped with the landraces (Fig. 3a, indicated with an arrow), while a restricted number of landraces either appeared as a first branching group to the modern varieties or they were nested within this larger cluster on both the tree and the PCA plot. The latter divided the landraces with mixed regional affiliations into three groups, one located on the upper right corner, consisting of mostly Hungarian landraces. The second admixed group was clustered along the center of the second axis, while the third group—also of mixed regional affiliation—was intercalated between the two clusters.

**Figure 4:**
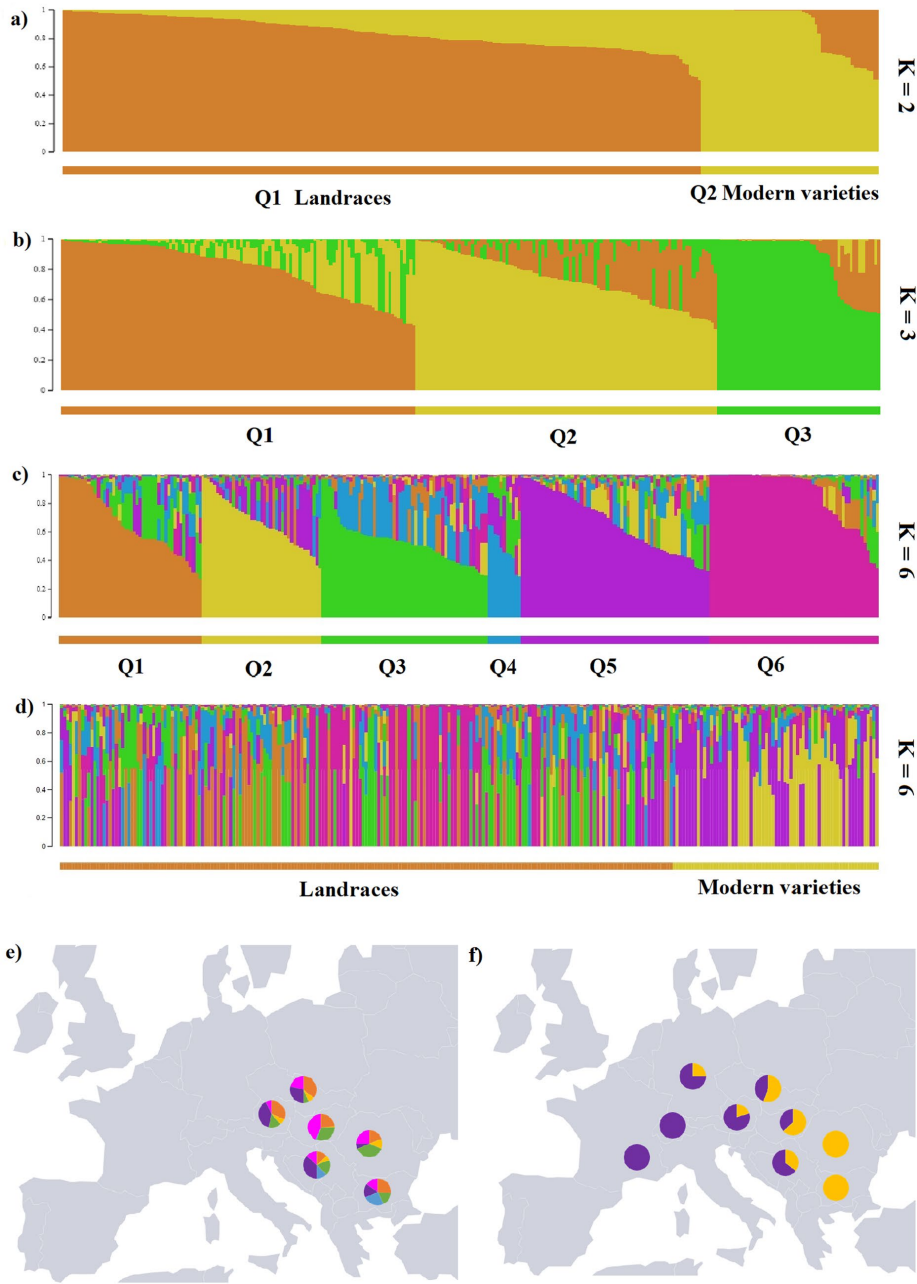
Patterns of admixture and population structure in the Central European historical wheat collection. (**a**) STRUCTURE model with *K* = 2 optimal clustering. Each accession is represented by an individual vertical line divided into *K* colored segments with heights according to genotype memberships in the clusters. The K = 2 divided the collection to landraces (*Q*1) and modern varieties (*Q*2). (**b**) At *K* = 3, the cluster of landraces was separated into two groups (*Q*1, *Q*2), and modern varieties were placed alongside closely related landraces (*Q*3). (**c**) Accessions and *K* = 6 clustering, vertical lines were given membership coefficients for each of the six clusters (*Q*1–6) if they had 50% or more participation in that group. (**d**) Plotted are STRUCTURE models with indicated *K* = 6 optimal clustering *Q* groups sorted according to chronological origin (landraces vs modern cultivars). (**e**) Pie charts depicting the average individual membership proportions (*Q*) in each of the six inferred ancestral groups identified by STRUCTURE analysis for each region from which landraces were gathered. The landraces were dominated by four ancestral groups (*Q*1, 3, 4, and 6) supporting the distinct separation of landraces from modern elite varieties. (**f**) Pie charts depicting the percentage memberships of modern wheat varieties. Ancestral group 5 dominates modern cultivars, while group 2 and 5 are mixed in Central Europe collection. The chart shows how the previously dominant four ancestral groups (*Q*1, 3, 4, and 6) in landraces were replaced by group *Q*2 and 5 over time in modern varieties.

A well-supported ML split (> 90%) can also be observed among the modern elite varieties, which divided the accessions into two major groups. This split was less prominent in the PCA, but loosely corresponded with the accessions clustering at the upper right corner of the plot and grouped closer to the first axis. The Bayesian cluster (STRUCTURE) analyses indicated a peak in the mean posterior probabilities LnP (K) at *K* = 6, with the lowest variance among replicates (Supplementary Fig. S2). The optimal number of clusters for the SNP dataset based on Δ*K* showed the highest peak at *K* = 2, with high peaks appearing at *K* = 3, and K = 6. This revealed that there are two genetically homogenous groups (*K* = 2) followed by three (*K* = 3) and six (*K* = 6) with the lowest variance among replicates in the Bayesian analysis. At *K* =3 the cluster of landraces was divided (*Q*1, *Q*2), and modern varieties were grouped together with closely related landraces (*Q*3) (Fig. 4b). Between 1930 and 1960 large part of the Hungarian wheat breeding was based on ‘Fleischmann’ (RMF70) and ‘Bankuti’ (RMF142) varieties^39^. They were appeared in the *Q*1 ancestral group well separated from the modern varieties. Between 1960 and 1980 ‘Bezostaya 1’ was the leading cultivar in Hungary^39^. One of its ancestors ‘Banatka’ (RMF21) grouped together with modern wheat varieties (*Q*3) in our analysis.

As our uneven sampling can bias the inferences on the number of Bayesian clusters, efforts were made to have comparable numbers of accessions from all countries evened across chronological groups. We carried out subsampling by removing closely related accessions using PCA clustering, and evaluated the Bayesian analyses based on the statistics described by Puechmaille et al.^40^. These statistics provided peaks for *K* values either at 2 (MedMedK and MedMeanK) or 6 (MaxMedK and MaxMeanK) and provided weaker support at 3. The *K* = 2 clustering divided the wheat collection into two differentiated groups, with a clear pattern of subdivision among the landraces and modern elite varieties (Fig. 4a). This grouping further corroborated the results obtained in the preceding ML and PCA analyses. We chose to describe *K* = 6, as we were interested in the overall clustering and differentiation among the entire collection at a regional level and this scenario was supported by subsampling. However, we have also overlaid the genetic origin information on the results (Fig. 4c,d). Thus, the average individual membership proportions (*Q*) of each region to the inferred clusters were divided into six distinct ancestral groups. The geographical distribution of the inferred Bayesian clustering was also investigated by projecting the inferred *Q* scores of the ancestral grouping on a map and on PCA clustering (Supplementary Fig. S3). Accessions were assigned membership to each of these six ancestral groups (*Q*1-6) if they had > 0.50 membership to that group.

Mean *Q* scores ranged from 0.001 to 0.995 for accessions across the six inferred clusters (Supplementary Table S6). The Bayesian clustering aligned with the patterns obtained in the ML and PCA analyses; it supported the distinct separation of modern elite varieties from landraces, and revealed further information about the fine-scale structure of the Central European wheat collection. Four ancestral groups (*Q*1, 3, 4 and 6) were predominant among the landraces, while *Q*2 and *Q*5 were mostly found among the modern elite varieties. Accessions with > 90% membership to ancestral group 5 were principally modern varieties, while group 2 was dominated by modern varieties from Romania and Bulgaria. This is in line with previous results of Winfield et al.^20^. Moreover, our ancestral group 5 possibly coincides with their ancestral group 2, mainly found in Western European varieties. The Central European landraces were shown to have admixed origins of four ancestral groups, where group 4 was the rarest—it was found in only 3% of the collection (> 0.50). However, some proportion (< 0.50) of this group was almost always found among the entire wheat collection. It had the highest proportion in a few landraces originating from the Balkans area (former Yugoslavia, ‘Dunav’, ‘Banja-Luka-6’: Bulgaria, ‘Sadovo-N-159’, ‘Stalinca’), and it was dominantly present only in one Hungarian landrace (‘Pitvaros’). Ancestral group 6, with accessions having > 90% membership, was exclusive to Central European landraces. It was highly dominant in the Hungarian landrace group (> 0.50), while it was found to be predominant (> 0.90) in 34% of the accessions.

### Recent loss of genetic diversity in modern elite varieties

**Figure 5:**
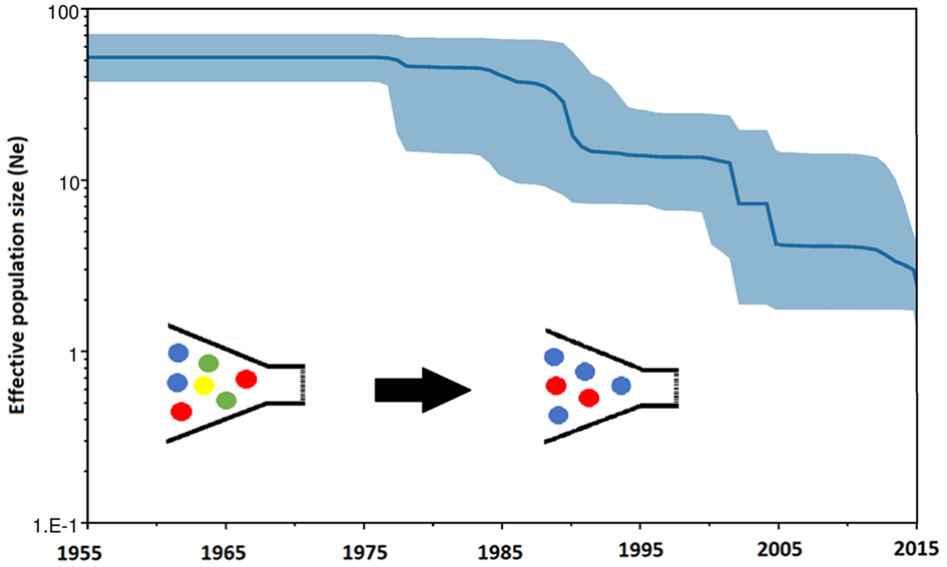
Bayesian Skyline plot (BSP) of the wheat collection depicting population size fluctuation over time. The vertical axis represents a time scale of calendar years between 1955 and 2015, while the horizontal axis represents changes in the inferred value of the effective population size over time (*N*e). The median estimate is shown as a black line, while the 95% highest posterior density intervals are shown in blue. The genetic erosion of the accessions due to breeding bottleneck is predominant in the figure.

We estimated the changes in genetic diversity through time and calculated the effective population sizes (*Ne*) of the Central European wheat collection between 1955 and 2015 (Fig. 5). The Bayesian Skyline Plot (BSP) analysis estimated *Ne* of the entire wheat collection to be 26.37 (95% HPD 36.64–18.15). A similarly small number (~ 30) was estimated by He et al.^41^ from a larger sample with exome sequencing, demonstrating the accuracy of our estimation despite sparser sampling. The results showed that the effective population size of wheat landraces kept stable at a plateau of 51.03 (95% HPD 69.53–36.85) during the 1980s until a bottleneck appeared and reduced the population size by ~ 21% (40.45, 95% HPD 65.22–10.56), with *Ne* remaining constant in the 1990s. A second bottleneck occurred in the 2000s that reduced the population size by ~ 28% (14.05, 95% HPD 30.51–7.12), dropping down to 1.72 (95% HPD 3.75–0.78) by 2015. The population size of modern wheat dramatically eroded by ~ 97% between 1955 and 2015.

The estimates of the effective population size for the modern elite varieties supported the findings of the ML tree that divided the collection into two groups associated with ancestral group 2 and 5 originating from the Balkans area, as inferred from the Bayesian cluster analysis. It should be noted that our sampling was limited among modern elite varieties; this effect can be seen in the wider HPD bonds obtained from the 2000s and the tighter values appearing for landraces from 1955 until the mid-1980s. Thus, a wider representative sampling might uncover a broader diversity that was not captured by our SNP array, and tighten the bonds of the HPD values. However, other studies conducted on a larger collection of wheat accessions showed that modern elite wheat has a small number of novel SNPs^20^, since breeding programs mainly used local landraces^1^. A good example of this pattern can be seen in the PCA grouping (Fig. 3a,d) where some landraces cluster together with the modern varieties.

## Discussion

Meeting the nutritional demand of the growing population with limited land use, decreasing water resources under the threat of climate change is the defining challenge of humanity in the 21st century^42^. Agriculture must simultaneously intensify, become more sustainable, and achieve greater resilience towards pests and climate change^7^. Environmental change is one of the worldwide difficulties confronting humankind today, as temperatures keep rising, setting off a large group of extraordinary climate fluctuations such as heat waves, dry seasons, and flooding^43^. Environmental changes are already affecting crop production levels and altering food security, this coupled together with the loss of genetic diversity in most crop species caused a post-domestication bottleneck^44^. Such changes are currently decreasing the worldwide yield of wheat, posing a major threat to global production that is estimated to decrease by ~ 6% for each 1 °C of temperature elevation^45–47^. Compensating such loss in production cannot be substituted by taking more land under cultivation without considering further serious effects on biodiversity loss and ecosystem services. Breeders will need to include as much positive genetic variation as they can to fulfill future needs in such scenarios. Genetic erosion caused by ‘green revolution’ and post-green revolution agro-practice adaption can be tackled by unlocking the genetic potential from historical landrace collections and wild relatives of wheat^20,48^. Many landraces kept in seed banks are not adequately characterized to attract breeders’ interest in their effective utilization. In most cases, the patterns of genetic diversity within and among such collections are unclear. However, comparison of the levels of nucleotide diversity in the modern elite varieties and landraces may provide valuable information for inferring the demographic history of wheat, the patterns of past breeding efforts, and signatures of selection events. Our genetic investigation of the 199 Central European landraces contrasted earlier studies reporting substantial duplication of germplasm accessions obtained from gene banks^49^. The landraces included in the present study were genetically distinct and their comparison with modern cultivars revealed a genetic signature that was defined by regions under selection. Mapping the positions of polymorphic markers along several agronomically important genes indicated that novel polymorphisms co-localize with these chromosome regions (Fig. 2). This indicates that during the modern breeding process these genome regions probably undergo intensive selection pressure.

Demographic events such as expansions or reductions can have long-term effects on the effective size of a population^50^. Thus, modeling the history of such demographic events in wheat can help to identify population differentiation by inferring past population-specific demographic changes. It has been shown that pure-line breeding and further agronomic improvement has resulted in population size reductions, typically referred to as genetic bottlenecks, in wheat^41,50,51^. Such bottlenecks help to explain the value of germplasm exchange and the use of landraces in modern breeding programs. In our comparative assessment based on the genetic origin and regional level using the Central European wheat landrace collection we revealed a split that lacks geographical division. However, the close clustering of landraces from the Balkans reaching the Black Sea coast (e.g. ‘Ahtopol’, ‘Goz’ and ‘Beljska’) with the modern elite varieties stood out in our analyses. Landraces appearing in the most basal position on the ML tree (Fig. 3) originated from this area, implicating that all modern elite varieties might share ancestry with these landraces. This reflects well the historical data showing that following World War II wheat varieties from the Adriatic, mostly from Italy, were imported by the former Yugoslav government with an aim to make the country self-sufficient in wheat production. The imported lines were used by local plant breeders resulting in the import of mutant *Rht8* alleles^52^. Another ML split also divided the landraces into two distinguished groups based on their basal groupings having varieties from the Balkans area in their first branching ancestry (Fig 3e, two-way arrow). Hence, these landraces might have had a greater impact on the genetic structure of the Central European landraces and modern wheat cultivars.

The geographical projection of the *Q* scores also revealed a change in the genetic composition of wheat accessions over time. According to this the Central European landraces were admixed from four ancestral groups, while the modern Central European wheat cultivars were composed of only two ancestral groups. There was a distinct East-West division among the collection, suggesting that elite varieties might have been selected from accessions originating from ancestral group 5 (Fig. 4). Dominant landraces (‘Fleischmann’, ‘Bankuti’) of the Hungarian wheat breeding between 1930 and 1960^39^ belonged to the *Q*3 ancestral group, showing only distant relationship to the modern wheat cultivars. ‘Bezostaya 1’—which carries the *Rht-B1e* insensitive allele—represented the leading Hungarian cultivar between 1960 and 1980, occupying almost 80% of the wheat-growing area in some years^39^. One of its ancestors, ‘Banatka’ (RMF21) ranked together with modern wheat varieties in the *Q*5 ancestral group demonstrating that this line may have contribute to modern breeding.

To further study this division, we evaluated the effective population size (*Ne*), which is an important population genetic parameter measuring the genetic diversity that can be maintained among the circumscribed collection. In this respect it defines the size of an ideal collection that would present the same amount of genetic drift as the collection of individuals under study; together with the mutation rate, it determines the number of alleles expected in collection. We detected a dramatic bottleneck in our investigated timeline (1955–2015), which might relate to the selection of a small number of founder lines for modern elite wheat breeding programs that relied on only a very limited number of parental lines in variety development (Fig. 5). This could be explained by the methods of plant breeders—they want to create new high-yielding varieties, and tend to make crosses among elite lines that have the highest likelihood of developing a new variety^53,54^.

The unprecedented availability of large-scale genomic resources, genome-wide association studies (GWAS) are now a valuable alternative to bi-parental QTL mapping defining with high precision the genetic architecture of the quantitative traits^55^. The application of GWAS in elite germplasm is generally limited to the identification of smaller-effect marker trait associations (MTAs), as major effect MTAs might have already become fixed within the modern wheat cultivars^56^. This limitation can be overcome by using a very diverse association panel composed of landraces and modern wheat cultivars.

Our investigation revealed an unexplored diversity within the Central European wheat landrace collection and pointed out the regions under a considerable selection during modern breeding. Our study showed the potential of genebank genomics to identify allelic diversity currently missing in breeding programs.

## Conclusions

Progress in genomics has resulted in new concepts and techniques that have the potential to improve the precision and efficiency of plant breeding. Reference genome assemblies, in conjunction with germplasm high-throughput SNP genotyping or sequencing, can help identify breeding targets that might help secure future food supplies. Significant advancements in plant genome sequencing explain how the availability of such resources, along with gene editing tools is transforming trait identification and modification operations. New techniques for breeding, such as genomic selection and speed breeding, may be able to overcome some of the constraints of traditional breeding. When genetics and genomics are integrated into breeding such as genotyping by sequencing, SNP genotyping, genomic selection, gene editing, rapid generation turnover, and haplotype-based breeding, the pace of genetic gains in breeding programs is projected to accelerate. Our work focusing on the genetic diversity of Central European wheat provided valuable information for understanding the relationships between landraces and modern elite varieties. We facilitated their characterization and determined their population structure and ancestral origins. Our results could enrich breeding strategies for future crop improvement through helping breeders to develop new varieties by reducing pre-breeding activities. Our data can be used, for example, to explore selective sweeps for any specific gene or chromosome region, analyze footprints defining divergence of landraces from distinct ecologies, or identify germplasm groups conserving allelic diversity missing in current breeding programs. The genomic data and analysis tools made public with this paper can assist wheat researchers to discover and use functional diversity that may be essential for meeting these challenges.

## Methods

### Plant material

The plant material consisted of 199 Central European winter wheat landraces originating from 6 countries (Austria, Bulgaria, former Czechoslovakia, Hungary, Romania, former Yugoslavia) and 67 modern winter wheat cultivars originating from 10 countries across the world (Austria, Bulgaria, former Czechoslovakia, France, Germany, Hungary, Romania, Switzerland, USA, former Yugoslavia). Part of the landrace collection originated from dissolved countries such as Yugoslavia (present day Bosnia and Herzegovina, Croatia, Kosovo, North Macedonia, Montenegro, Serbia and Slovenia) and Czechoslovakia (Czechia and Slovakia). The landraces were collected during 1950-60 and conserved in the Gene Bank collection of the Centre for Plant Diversity (NÖDIK) Tápiószele, Hungary. The modern wheat cultivars used in the present study were registered between 1970 and 2015. The description of the accessions is detailed in the Supplementary Table S5. Within the modern cultivars we defined ‘Western varieties’ as a subgroup, which included varieties from Austria, Germany, France, Switzerland and the USA. The plant material used in our study is not regulated by CITES and IUCN, and sampling followed national and international guidelines for germplasm management outlined by FAO. Before using the landraces for any analysis, the lines were homogenized three times via using the single seed descent (SSD) methodology.

### SNP genotyping

DNA was extracted from 6 weeks old seedlings using the DNeasy Plant Mini Kit (Qiagen, Hilden, Germany) according to the manufacturer’s instructions. SNP genotyping was performed by TraitGenetics GmbH (http://www.traitgenetics.com/en/), using a 20K Illumina SNP chip, which represents a subset of markers from the 90K SNP array and the 35K Axiom Wheat Genotyping Breeders’ Array^19,57^. SNPs with greater than 10% missing values and 5% heterozygosity were removed from the subsequent analysis, which left a set of 15,808 high quality SNP markers.

### Analysis of genetic diversity

To determine which landraces are particularly polymorphic compared to the modern varieties, we calculated the genotypic scores for the accessions and the number of additional polymorphisms using the SNP call function of Geneious Prime (Biomatters Ltd, Auckland, New Zealand) with default settings. Polymorphic sites among landraces, as well as their chromosomal positions and allele frequencies were determined through a comparison with all modern varieties, and separately with a small subset consisting of only modern varieties originating from Western Europe or the USA. The average number of nucleotide differences per site (nucleotide diversity; π, Jukes and Cantor 1969)^58^ was calculated with DnaSP v6^59^ using default settings. This was done for each chromosome based on size, as described by Šafář et al.^36^. The number of SNPs and marker density of all wheat chromosomes based on ‘MV Ménrót’ (RMF209) reference were calculated with Geneious Prime. ‘MV Ménrót’ is one of the leading cultivars in Hungary and it is used as a general standard by the National Food Chain Safety Office (Hungary) during the plant variety registration process. The position of several genes of great agronomic importance^60,61^ were plotted alongside polymorphic sites to identify co-localizing regions. Circular ideograms from the calculated values were created with shinyCircos^62^. General descriptive statistics of the SNPs such as the heterozygosity index (*H*), polymorphic information content (*PIC*) and discriminatory power (*D*) was calculated with iMEC^63^.

### Haplotype construction and population structure analysis

Haplotype blocks were constructed for each chromosome separately using HAPLOVIEW 4.2^64^. Block construction was done based on the confidence interval algorithm of Gabriel et al.^65^ that considers LD(D’) among the markers or loci within and outside a proposed block. Principal component analysis (PCA) was used to summarize patterns of variations among the wheat accession. The analysis was carried out with Tassel v5 according to Bradbury et al.^66^ excluding SNPs with minor allele frequency < 0.01. Relationships among wheat varieties were also inferred using a maximum likelihood (ML) approach implanted in IQ-TREE v1.6.12^67^. The general time reversible (GTR + F) nucleotide evolutionary model with direct base frequency counts was chosen as best-fitting for the dataset inferred with the -TESTMERGEONLY and -AICc options in the built-in ModelFinder^68^. The analyses were performed using the ultrafast bootstrap approximation (UFBoot2)^69^ with 1000 replicates to provide relatively unbiased bootstrap estimates under mild model misspecifications reducing computing time and achieving unbiased branch supports. Unrooted trees were visualized with FigTree v1.4.4^70^.

For the analysis of population structure, a model-based Bayesian cluster analysis was performed using STRUCTURE v2.3.4^71^. Despite being orders of magnitude slower, STRUCTURE was chosen as an analysis platform since it performs well with polyploid data and provides an unbiased inference when differentiation is weak^72^. We used the *admixture* model with an inner *alpha* incorporating prior pedigree-based data assuming for the relative mixed ancestry of the accessions from multiple sources. The best fitting number of assumed clusters (*K*) ranging from 1 to 10 was evaluated performing 10 independent Markov chain Monte Carlo (MCMC) runs of 1 × 10^6^ iterations following a burn‐in period of 1 ×10^5^ steps for each value of *K*. The best *K* was chosen based on the estimated membership coefficients (*Q*) for every individual in each cluster. The best fitting number of assumed clusters (*K*) was estimated using lnP(D∣K)^71^ and Δ*K* rate change in the log probability of data between consecutive K values^73^, as implemented in StructureSelector^74^. The results of STRUCTURE are greatly affected by sample size, since the program accounts for the most salient variation. Thus, we also used the metrics MedMeaK, MaxMeaK, MedMedK and MaxMedK^40^ derived from the posterior probability for each K across multiple MCMC replicates. Given that unbalanced sample sizes among landraces and modern varieties could hamper the recovery of genetic clusters, we subsampled our dataset in order to maintain sample sizes as even as possible using the same model assumptions and parameters. We then used CLUMPAK v1.1.2^75^ to find the best alignment of the results across the range of *K* values as implemented in StructureSelector and visualized the results using STRUCTURE PLOT v2^76^.

### Bayesian skyline prediction

The historical demographic changes of wheat varieties were inferred from the estimate of effective population size (Ne) over time in order to explore temporal fluctuations. This method is a non-parametric approach developed for the inference of past population size changes from genetic data built on a piecewise-linear model of Markov Chain Monte Carlo (MCMC) probability distribution sampling to reconstruct demographic history. The SNP data was loaded to BEAUTi v.2.3.3. to set parameters and specific model criteria. The years when each wheat variety was bred were extracted from archival records and were set as tip calibration points. The initially chosen best fitting GTR nucleotide substitution model was used for Ne estimations, additionally allowing for rate heterogeneity among sites by setting the Gamma Category Count to 4 and estimating the Gamma distribution shape parameter while leaving the substitution rate fixed, which allowed the clock rate to estimate the number of substitutions per site per year. Coalescent Bayesian Skyline was selected as a tree-prior, which divided the time between modern and old landraces into segments estimating Ne for each branching time in the tree. The number of parameter dimensions was set to four, allowing Ne to change three times between the root and present day. Ten Markov chains were run for 1 × 10^7^ generations using BEAST v2.3.2^77^ and were sampled every 1000 steps, with the first 1 × 10^6^ samples discarded as burn-in. Runs were analyzed using Tracer v1.6, and convergence was verified by assessing effective sample sizes (ESS) of all parameters. Independent runs were combined in LogCombiner v2.3.2.

### Ethical standards

On behalf of all co-authors, the corresponding author states that the work described is original, previously unpublished research. All the authors listed have approved the manuscript.

### Statement of plant material collection

The plant material used in our study is not regulated by CITES and IUCN. Our sampling followed national and international guidelines for germplasm collection outlined by FAO. Plant material was obtained under the permission of a Standard Material Transfer Agreement (SMTA) of the International Treaty on Plant Genetic Resources for Food and Agriculture.

## Supplementary Information

Supplementary Figure S1.

Supplementary Figure S2.

Supplementary Figure S3.

Supplementary Table S1.

Supplementary Table S2.

Supplementary Table S3.

Supplementary Table S4.

Supplementary Table S5.

Supplementary Table S6.

## Data Availability

The datasets generated and analyzed during the current study are available from the corresponding author on reasonable request.
