# Supplementary figures and images for: Exploring the legacy of Central European historical winter wheat landraces
Source: Sci Rep. 2021 Dec 13;11:23915. doi: 10.1038/s41598-021-03261-4 (PMC8668957; doi:10.1038/s41598-021-03261-4)

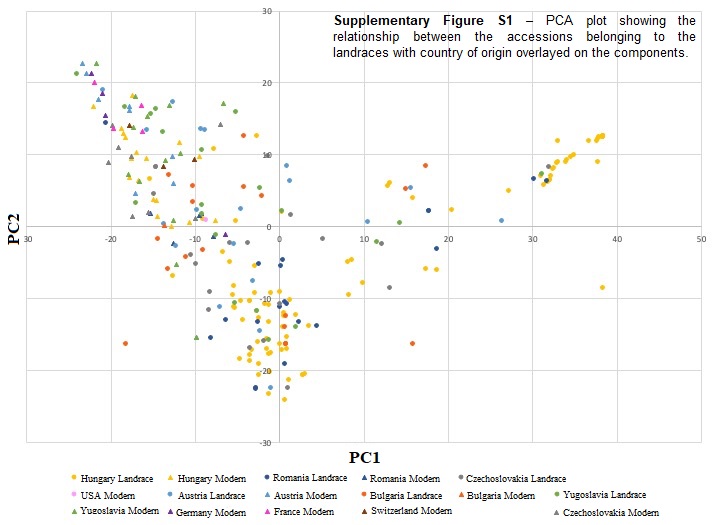

Supplement: Supplementary file 1 — Supplementary Figure S1. [file 41598_2021_3261_MOESM1_ESM.jpg]

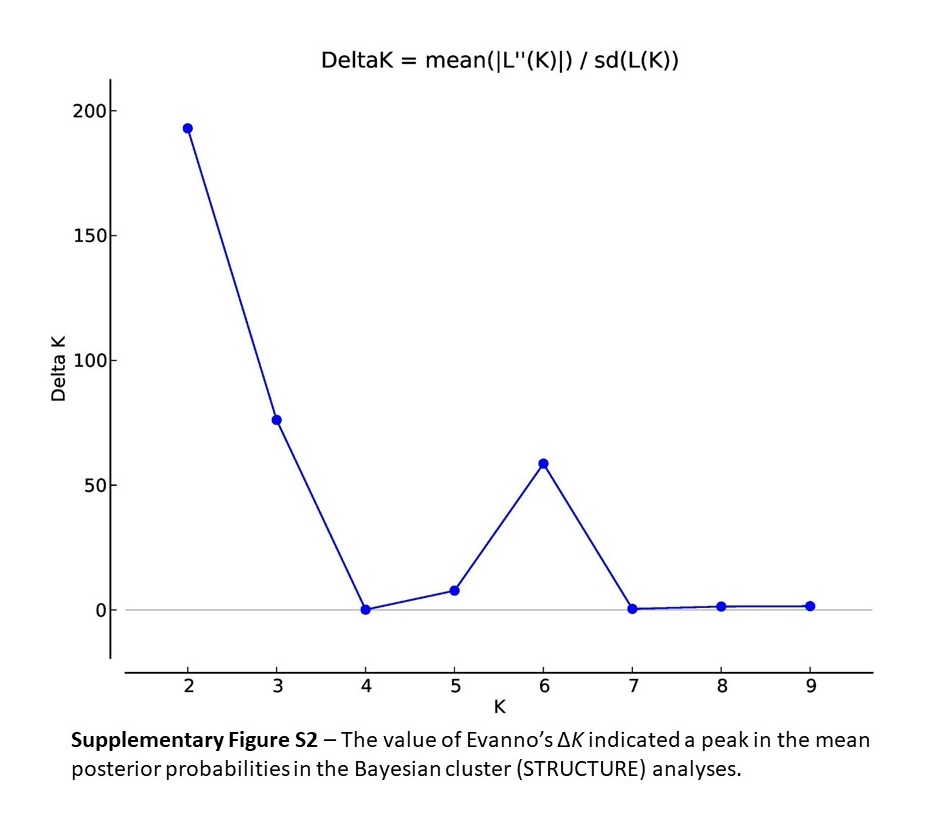

Supplement: Supplementary file 2 — Supplementary Figure S2. [file 41598_2021_3261_MOESM2_ESM.jpg]

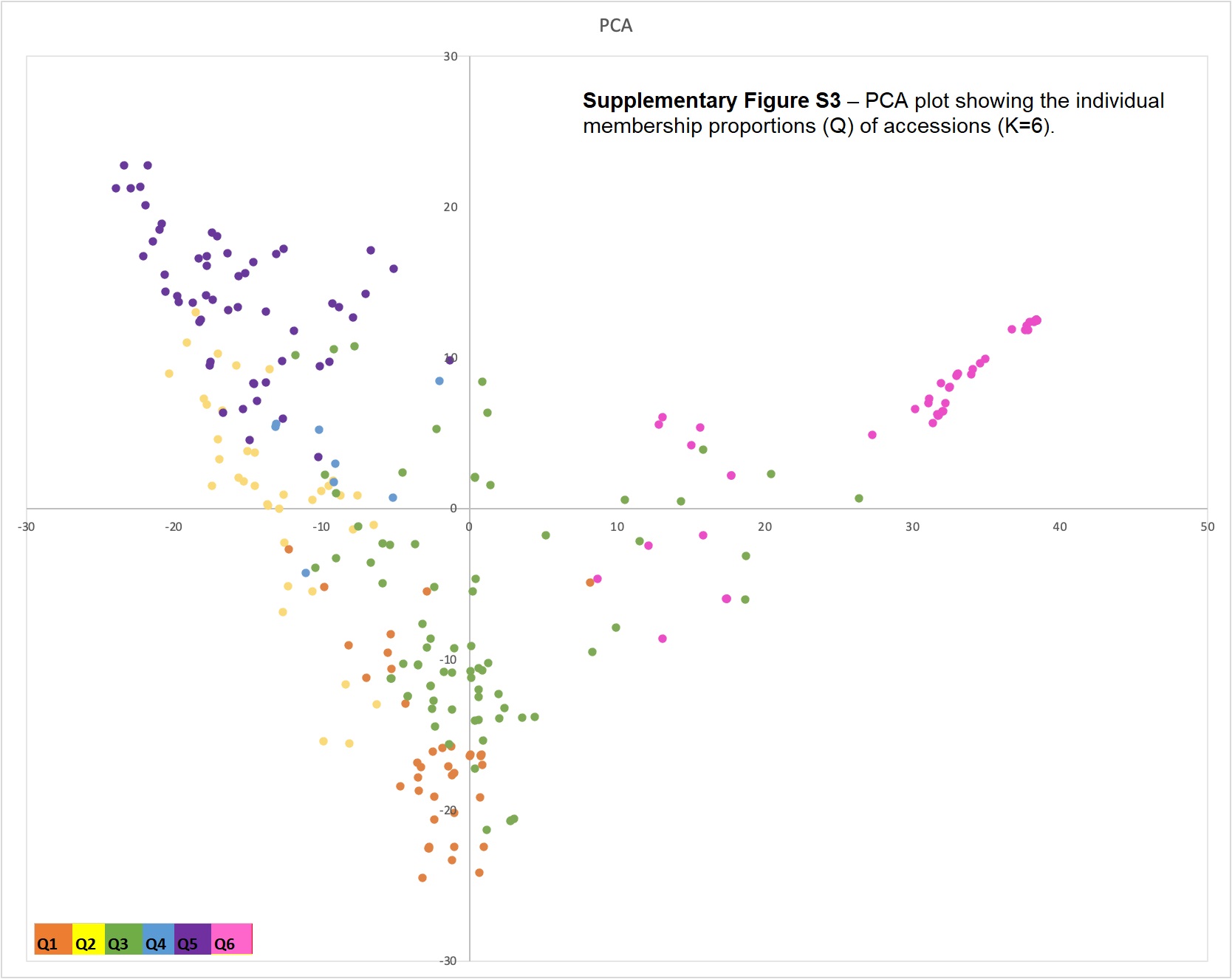

Supplement: Supplementary file 3 — Supplementary Figure S3. [file 41598_2021_3261_MOESM3_ESM.jpg]
